# Two-dimensional Tunable Dirac/Weyl Semimetal in Non-Abelian Gauge Field

**DOI:** 10.1038/s41598-019-54670-5

**Published:** 2019-12-06

**Authors:** Yaowu Guo, Zhi Lin, Jia-Qiang Zhao, Jie Lou, Yan Chen

**Affiliations:** 1 Department of Physics and Optoelectronic Engineering, Weifang University, Weifang, 261061 China; 2 Department of Physics and State Key Laboratory of Surface Physics, Fudan University, Shanghai, 200438 China; 3 School of Physics and Materials Science, Anhui University, Hefei, 230601 China; 4 Collaborative Innovation Center of Advanced Microstructures, Nanjing University, Nanjing, 210093 China

**Keywords:** Superconducting properties and materials, Topological matter, Physics

## Abstract

Three-dimensional(3D) Weyl semimetal(WSM) with linear energy spectra has attracted significant interest. Especially they have been observed experimentally in several solid materials with the breaking of inversion symmetry. Here we predict a new family of particle-hole(${\mathscr{C}}$) invariant 2D WSMs in the non-Abelian gauge field, which can emerge in the low energy bands being close to Fermi energy (dubbed Weyl-I) and the high energy bands being away from Fermi energy (dubbed Weyl-II), only when the time-reversal symmetry(${\mathscr{T}}$) of the 2D Dirac semimetal is broken in the presence of in-plane Zeeman fields. Moreover, a 2D Dirac node can split into a pair of Weyl nodes showing the same Berry phase, and the 2D WSM, being protected by ${\mathscr{T}}$ symmetry, exhibits four Weyl-I nodes, whose energies are invariant with the variation of the magnetic field. The corresponding Fermi velocity and Berry connection have been calculated. Based on the 2D WSMs, we also examine inhomogeneous pairings of attractive Fermi gases and find a new kind of the LO states with the beat frequency. This 2D WSM provides a realistic and promising platform for exploring and manipulating exotic Weyl physics, which may increase the experimental feasibility in the context of ultracold atoms.

## Introduction

The search for the topology of band structures has become an important subject in modern condensed matter physics. Notable examples are topological insulator and superconductivity^1,2^, which are gapped in bulk but possess gapless surface states on the boundary of the system. Recently, theoretical and experimental developments of topological semimetals have generalized topological phases from gapped to gapless system^3–12^. In particular, the 3D WSM will emerge with a broken time-reversal or inversion symmetry, which has pairs of Weyl points in bulk. Near such a Weyl point, the energy spectrum is linear along three momentum dimensions and described by Weyl Hamiltonian. A pair of Weyl points with opposite chiralities can be connected by open Fermi arc on the surface of the 3D system^3–5^. Different from this family of 3D WSMs, dubbed type-I WSM, with a point-like Fermi surface, type-II WSM holds a novel type of structured Weyl point^13^ that exists at the contact point of the electron and hole pockets of the Fermi surface^14–18^. Due to the gapless topological structures of type-I and II WSMs in bulk, they exhibit many novel properties, such as chiral anomaly, anomalous magnetic effects and so on^19–23^. Significant experimental progress has been reported on two types of WSMs in solid-state materials^2,10–12,14–18^ and artificial systems^24^. In all the previous experimental works on type-I or type-II WSMs, Weyl nodes in pairs with opposite chiralities are found in the 3D systems. The 2D WSM has been theoretically explored in the low energy effective theory^25^, and quasiparticle spectra of the superconductor in the heterostructure^26^, but theoretical schemes and experiments about the emergence of 2D time-reversal breaking WSMs in the single-particle spectra of the true 2D systems do not exist.

On the other hand, the experimental realization of the 1D and 2D spin-orbit couplings in ultracold atomic gases has propelled these artificial systems as promising platforms for discovering novel topological states of matter^27,28^. To realize the WSM, a lot of experimental schemes focus on 3D single-particle spectra^29–32^, the 3D quasiparticle spectra^33^, and equivalent 3D systems with an artificial dimension by parameters of the model or the internal degrees of freedom of the system^34^. All these proposals have increased the difficulty in the controllability of experiments. It is worth noting that a synthetic external Abelian and non-Abelian gauge field coupled to neutral atoms have been generated in ultracold atomic gases experimentally^35–37^. For the non-Abelian gauge field^38,39^, it can simulate various relativistic quantum field theories, where the energy bands may display various kinds of singularities, such as Dirac points. A Dirac point in the 3D system may split into two Weyl points when inversion or time-reversal symmetry (TRS) is broken, and the Dirac semimetal becomes the WSM^40^. A natural question to be asked is whether a feasible and straightforward scheme that a 2D Dirac point in the single-particle spectra may split into two Weyl points in the mere 2D lattice system exists.

In this article, we report the emergence of the 2D Weyl points in the single-particle spectra with the non-Abelian Gauge Field and the in-plane Zeeman field. Only when the non-Abelian Gauge Field exists, can the system show two Dirac points in the first RBZ, similar to the Dirac cones of the honeycomb lattice. When increasing in-plane Zeeman field strength, each fourfold degenerate Dirac point is broken into double degenerate Weyl points. Four Weyl points can be found at the half-filling, and it only changes the position of the momentum with the change of the in-plane Zeeman field, dubbed Weyl-I. Meanwhile, two groups of Weyl points can be found at the positive and negative energies, respectively, dubbed Weyl-II, when deviating from the half-filling. Remarkably, the size of the positive and negative energy of these Weyl-II points is consistent with the size of the in-plane Zeeman field strength, and the variation of the Zeeman field only modulates their energy positions. Based on this WSM, a novel FFLO pairing of attractive Fermi gases with the beat phenomenon can be formed on pairs of the Fermi surfaces of the system.

## Model Hamiltonian

To explore the WSMs, the non-Abelian gauge field can be considered via the Peierls substitution **A**^±^ = (±*ασ*_*x*_, $\mp b{s}_{y}$) in the 2D system. Due to the position dependence of the non-Abelian gauge field, we need AB sublattice, to which the upper and lower symbols are corresponding respectively, and *σ*_*x*_ and *σ*_*y*_ are the Pauli-spin matrices. Combined with this non-Abelian gauge field, we consider a tight binding model for Fermi gases trapped in a 2D square lattice, whose Hamiltonian is written as

1 $$H=-\,t\sum _{i\in A(B)}\,{c}_{i\sigma }^{\dagger }{U}_{ij,\sigma \sigma ^{\prime} }^{+(-)}{c}_{j\sigma ^{\prime} }+{h}_{x}\sum _{i\sigma }\,{c}_{i\sigma }^{\dagger }{c}_{i\bar{\sigma }}-\mu \sum _{i\sigma }\,{c}_{i\sigma }^{\dagger }{c}_{i\sigma }$$

where *t* is the nearest-neighbor hopping energy, *h*_*x*_ is in-plane Zeeman field, *μ* is chemical potential, $\bar{\sigma }$ represents the opposite direction of the spin *σ*, and ${c}_{i}^{\dagger }$ and *c*_*i*_ create and annihilate a fermion at lattice site *i* respectively. The unitary operator *U* can be written as ${U}_{x}^{\pm }={e}^{\pm ia{s}_{x}}$ and ${U}_{y}^{\pm }={e}^{\mp ib{s}_{y}}$. For simplicity, we choose *t* = 1 as the energy unit and *α* = *β* = *π*/4.

To illustrate the emergence of Weyl points, the whole Hamiltonian under the period boundary condition may be rewritten in momentum space with the new basis: Ψ_*k*_ = $({\hat{A}}_{k\uparrow },\,{\hat{A}}_{k\downarrow },\,{\hat{B}}_{k\uparrow },\,{\hat{B}}_{k\downarrow })$.

2 $${H}_{k}={h}_{t}{\tau }_{x}\otimes {\sigma }_{0}+{h}_{so}^{x}{\tau }_{y}\otimes {\sigma }_{x}+{h}_{so}^{y}{\tau }_{y}\otimes {\sigma }_{y}+{h}_{x}{\tau }_{0}\otimes {\sigma }_{x}$$

where *h*_*t*_ = −2*t*(cos *α*cos *k*_*x*_ + cos *β*cos *k*_*y*_), and ${h}_{so}^{x}$ = 2*t*sin *α*cos *k*_*x*_, ${h}_{so}^{y}$ = −2*t*sin *β*cos *k*_*y*_. *τ* and *σ* is Pauli matrices acting on AB sublattices and spin components respectively. Here, the Hamiltonian has the inversion-like symmetry(${\mathcal I}$) and the time reversal symmetry(${\mathscr{T}}$) in the absence of *h*_*x*_^41^: ${\mathcal I} H({\bf{k}}){( {\mathcal I} )}^{-1}=-\,H(\,-\,{\bf{k}})$, ${\mathscr{T}}H({\bf{k}}){({\mathscr{T}})}^{-1}=H(\,-\,{\bf{k}})$, with ${\mathscr{T}}={\tau }_{0}\otimes {\sigma }_{y}{\mathscr{K}}$, ${\mathcal I} ={\tau }_{x}\otimes {\sigma }_{z}$ and ${\mathscr{K}}$ being the complex conjugate operator, and these two kind of symmetries guarantee that the spectrum degenerates four-fold for each gapless touching point, Dirac point. When increasing in-plane Zeeman field, the ${\mathscr{T}}$ symmetry is broken, and each fourfold degenerated Dirac point is broken into a pair of two-fold degenerated Weyl points. At this moment, the Hamiltonian still has the particle-hole(${\mathscr{C}}$) symmetry. It plays a crucial role here because a pair of Weyl points with opposite momenta(±**k**) have the same topological characteristics, and each Weyl point will not annihilate until they melt with the Weyl point with opposite topological characteristics. The neutrality of topological charge in the entire *k* space means the other two Weyl points with opposite topological characteristics exist at some other momenta. For one Weyl point, the presence of topological characteristics allows the definitions of the Berry phase^33^ as follows:

3 $${n}_{w}=\frac{1}{\pi }\mathop{\oint }\limits_{l}\,{{\mathscr{A}}}_{{\boldsymbol{k}}}\cdot {\rm{d}}{\boldsymbol{k}}$$

where ${{\mathscr{A}}}_{{\boldsymbol{k}}}$ is the Berry connection of the energy band holding the Weyl point, and the loop *l* surrounds the Weyl point.

## The Emergence of Weyl Points

**Figure 1:**
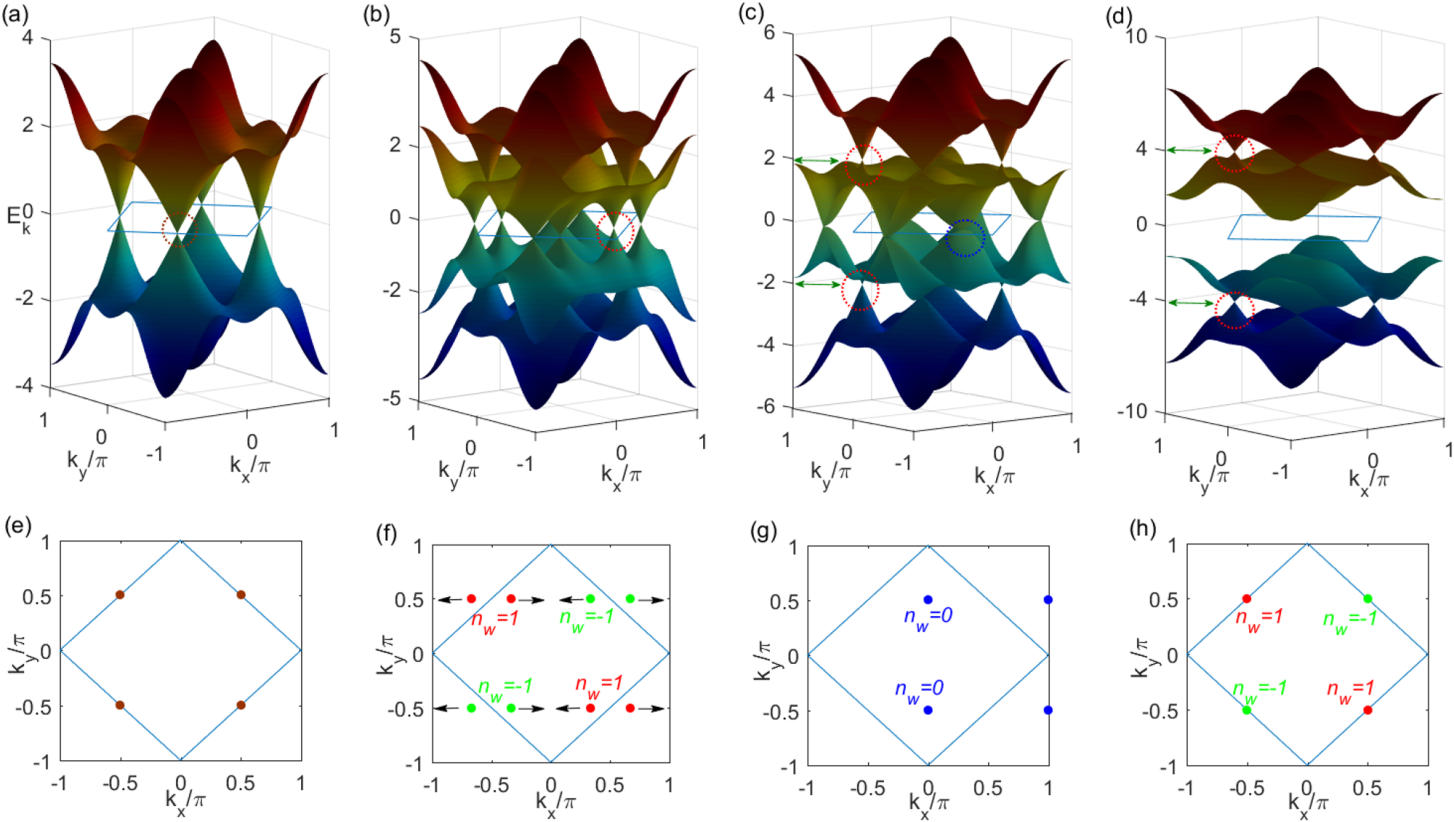
Features of 2D Dirac and Weyl semimetals from the energy spectrum as a function of in-plane magnetic field *h*_*x*_. (**a**) The energy spectrum of the Dirac semimetal with *h*_*x*_ = 0. (**b**) The WSM with *h*_*x*_ = 1. (**c**) The merging of Weyl points with *h*_*x*_ = 2. (**d**) Weyl-II points in the gapped system with *h*_*x*_ = 4. (**e**–**g**) The corresponding Dirac and Weyl-I points on the Fermi surfaces for *h*_*x*_ = 0, 1, 2 accordingly. (**h**) Weyl-II points on the two Fermi surfaces with the chemical potential *u* = ±*h*_*x*_. Winding numbers of red and green points are 1 and −1 respectively.

In addition to the symmetry analysis, we demonstrate the emergence of Weyl points from the energy spectrum. The quasiparticle energy of *H*(**k**) read ${E}_{k}=\pm \,\sqrt{{h}_{so}^{y2}+{({h}_{x}\pm \sqrt{{h}_{t}^{2}+{h}_{so}^{x2}})}^{2}}$. In Fig. 1, we further depict the band structures of some representative parameters *α* = *β* = *π*/4 for Dirac and Weyl Semimetal. In two dimensional momentum space, without *h*_*x*_, four Dirac points can be found at $(\pm \frac{\pi }{2},\pm \,\frac{\pi }{2})$ such as in Fig. 1(a), In Fig. 1(e), four dots display Fermi points of the Dirac semimatal. In Fig. 1(b), when increasing *h*_*x*_, Weyl points can emerge in pairs with the opposite chiralities, corresponding to *n*_*w*_ = ±1 in Fig. 1(f). The locations of Weyl points can satisfy the conditions of cos $({k}_{x})=\pm \,\frac{{h}_{x}}{2}$ and ${k}_{y}=\frac{\pi }{2}$ near the half filling, dubbed Weyl-I, whose momenta are variant with the change of *h*_*x*_. Its locations can be tuned easily just by varying *h*_*x*_. Meanwhile, between the higher two energy bands or the lower two energy bands, four Weyl points can also be found at $(\pm \frac{\pi }{2},\pm \,\frac{\pi }{2})$ respectively, in Fig. 1(h), dubbed Weyl-II, whose momenta are invariant with the change of in-plane Zeeman field *h*_*x*_ strength, and energies are equal to the size of *h*_*x*_, as in Fig. 1(d). As *h*_*x*_ changes from weak to strong, Weyl-II points are very robust and cannot merge. In contrast with Weyl-II points, as *h*_*x*_ ≈ 2, two Weyl-I points with the opposite Berry phase will merge, and the total Berry phase becomes zero in Fig. 1(c,g). It is noteworthy that the slight magnetic field can lead to the emergence of the 2D WSM, and it increases the feasibility of the experiment on the ultracold atom.

For any Weyl point, the location of the momentum is at *k*_*w*_. To obtain the low-energy effective Hamiltonian^42^, we have ${V}_{{k}_{w}}^{\dagger }H({k}_{w}){V}_{{k}_{w}}=({E}_{1},{E}_{2},0,0)$ and ${\Phi }_{{k}_{w}}={V}_{{k}_{w}}^{\dagger }{\Psi }_{{k}_{w}}$ by diagonalizing *H*(*k*_*w*_). Using the vector *V*_*kw*_, we can separate 4 × 4 *H*(*k*) matrix into 2 × 2 blocks as:

4 $${\tilde{H}}_{k}={V}_{{k}_{w}}^{\dagger }H(k){V}_{{k}_{w}}=(\begin{array}{cc}{H}_{h} & {H}_{c}\\ {H}_{c}^{\dagger } & {H}_{l}\end{array})$$

and ${\Phi }_{k}={V}_{{k}_{w}}^{\dagger }{\Psi }_{k}=({\phi }_{h},{\phi }_{l})$, where *H*_*h*_ and *H*_*l*_ are the high-energy and low-energy components, *H*_*c*_ is coupling between two components. We can integrate out the high-energy component *ϕ*_*h*_, and the effective two bands Hamiltonian can be obtained as ${H}_{eff}={\phi }_{L}^{\dagger }[{H}_{l}-{H}_{c}^{\dagger }{H}_{h}^{-1}{H}_{c}]{\phi }_{l}$. Moreover, we can get the low energy effective hamiltonian by expanding *H*_*eff*_ in the vicinity of this Weyl point, which is described by Weyl hamiltonian ${H}_{w}=\sum _{i(j)=x,y(y,z)}\,{v}_{ij}{q}_{i}{\sigma }_{j}$, where **q** = **k** − **k**_*w*_ is the displacement vector from Weyl point, *v*_*ij*_ is a 2 × 2 matrix. Althoug*h h*_*x*_ breaks ${\mathscr{T}}$ symmetry of global hamiltonian in Eq. (2), for this low-energy effective Weyl hamiltonian, the joint symmetry of ${\mathscr{P}}{\mathscr{T}}$ is allowed by choosing ${\mathscr{P}}={\sigma }_{x}$ and ${\mathscr{T}}=-\,i{\sigma }_{y}\hat{{\mathscr{K}}}$ with the ${{\mathbb{Z}}}_{2}$ topological charge^41^:${\mathscr{P}}{\mathscr{T}}H({\bf{k}}){({\mathscr{P}}{\mathscr{T}})}^{-1}=H({\bf{k}})$. The topological characteristic of the ${{\mathbb{Z}}}_{2}$ Weyl point can also be defined as *χ* = sign (det [*v*_*ij*_]), and the calculation shows that the general relation of these two invariants *χ* and *n*_*w*_ is *χ* = −*n*_*w*_, both of which directly reflect the topological characteristics of the Weyl point together.

**Figure 2:**
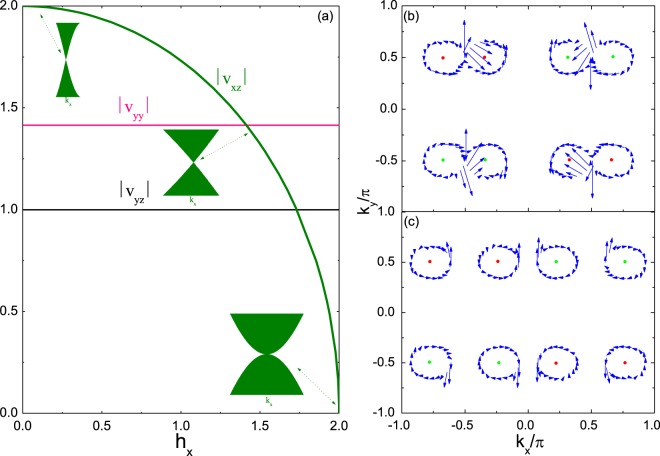
The low-energy effective Fermi velocity and Berry connection of the winding number. In (**a**) the components of Fermi velocities as a function of *h*_*x*_ under the low-energy effective theory. In (**b**) 2D vectorgraph of Berry connections around different Weyl points at the zero energy with *h*_*x*_ = 1 and *μ* = 0, and (**c**) *h*_*x*_ = 1.5 and *μ* = 0, and the arrows represent the direction of Berry connections. For red and green points as in Fig. 1.

To better explore the tunability of the Weyl point under the magnetic field, we calculate the components of the effective Fermi velocity(*v*_*F*_) as a function of *h*_*x*_ under the low-energy effective theory in Fig. 2(a). We can find that *v*_*yy*_ and *v*_*yz*_ components are all invariant with increasing *h*_*x*_. *v*_*xz*_ decreases with the increase of *h*_*x*_. When *h*_*x*_ is close to 2, *v*_*xz*_ = 0, linear dispersions of *E*_*k*_ are dropped, and Weyl points are transformed into normal Fermion particles. All of these characteristics are reflected by insets of *E*_*k*_(*k*_*x*_). By increasing *h*_*x*_, Berry connections between two closely spaced Weyl points demonstrate strong interference. Here, by taking the constant phase factor of the wave function, the Berry connection becomes gauge-invariant. Berry connections of two Weyl points with the same Berry phase intensively scatter and cancel each other in Fig. 2(b). In Fig. 2(c), when two Weyl points with opposite Berry phases close, Berry connections reinforce between them. The emergence of all these phenomena depends only on the modulation of the magnetic field.

## Detection of the WSM using the Bloch Zener Oscillation

**Figure 3:**
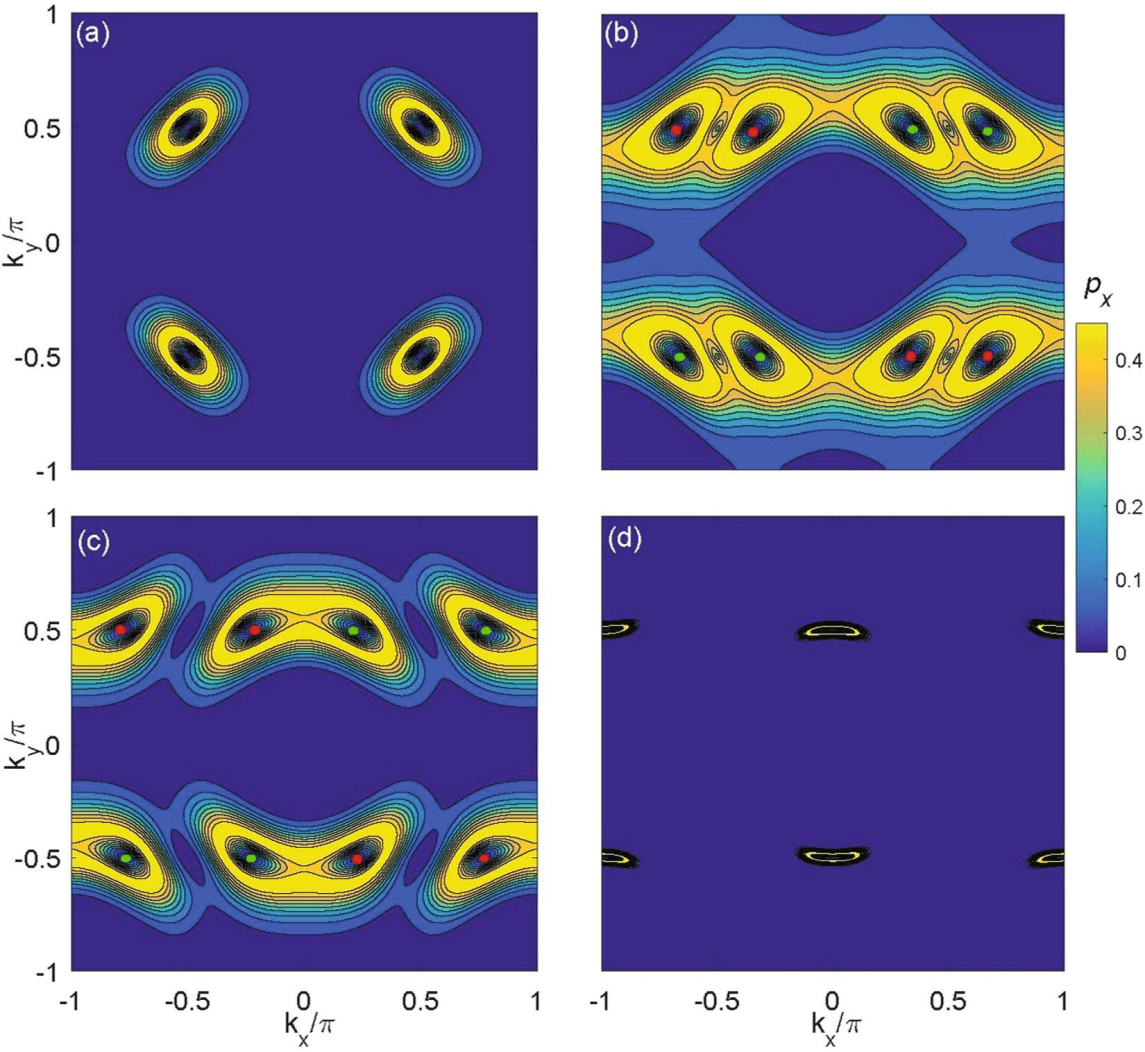
The 2D momentum distribution of the transfer fractions *p*_*x*_ for different *h*_*x*_. (**a**) The qusimomentum distribution of *p*_*x*_ for Dirac points with *h*_*x*_ = 0.0. The qusimomentum distribution of *p*_*x*_ for Weyl points with (**a**) *h*_*x*_ = 1.0, (**b**) *h*_*x*_ = 1.5, and (**d**) *h*_*x*_ = 1.99. For (**a**–**c**), the distribution of *p*_*x*_ exhibits the ring profiles. The dip inside a ring labels the location of the Dirac point or the Weyl point. Other parameters are *t* = 1 as energy unit and *F* = 1, For red and green points as in Fig. 1.

Landau-Zener tunneling from the lower to the upper band has revealed that the atomic fraction tunneling can monitor the band-touching points to the excited band after a cycle of Bloch oscillations^43^. Especially, recent experiments utilize the Bloch Zener Oscillation technique to explore the two-band system featuring Dirac points^44^. This technique is also extended to detect Weyl points in the 3D lattice system^31,45^. Below, we utilize this technique to explore the case of the 2D WSM, in which the transfer fraction can be written as

5 $${p}_{x}({k}_{x},{k}_{y})=2{P}_{LZ}({k}_{x},{k}_{y})[1-{P}_{LZ}({k}_{x},{k}_{y})],$$

where ${P}_{LZ}={e}^{-{\pi }{E}_{+}^{2}({k}_{x},{k}_{y})/{v}_{x}F}$ is Landau-Zener transition probability. *E*_+_(*k*_*x*_, *k*_*y*_) denotes the difference of the energy locating at (*k*_*x*_, *k*_*y*_) when Landau-Zener transitions arise, *v*_*x*_ is velocity near Weyl points, and *F* is an external constant force. When *F* is applied in the 2D WSM along *x* direction, atoms in the ground state can be pushed and move along *x* direction, whose process may be reflected by the 2D quasimomentum distribution of the transfer fractions *p*_*x*_. For different *h*_*x*_, results of *p*_*x*_ can be displayed in Fig. 3. In Fig. 3(a) with *h*_*x*_ = 0, the distribution of the transfer fraction *p*_*x*_ exhibits four the ring-type profiles in the first BZ. For any ring-type profile, the position of dip (*p*_*x*_ = 0) inside it symbols the location of the Dirac point. In Fig. 3(b) with *h*_*x*_ = 1, *p*_*x*_ also exhibits four the ring-type profiles in the first RBZ and positions of red and green points (*p*_*x*_ = 0) symbol locations of the Weyl points. The strength *p*_*x*_ between any two Weyl points with the opposite Berry phases weakens and forms a vortex. When increasing *h*_*x*_ to 1.5, two pairs of Weyl points with opposite Berry phases get close to each other in Fig. 3(c), the strength of *p*_*x*_ between them reinforces. The strength of *p*_*x*_ exactly reflects the distribution of Berry connections around Weyl points in Fig. 2(b,c). In Fig. 3(d), when *h*_*x*_ = 1.99, Weyl points with opposite Berry phases can move together and merge. The emergence indicates the critical point of the phase transition, which can be identified by this Bloch Zener Oscillation method. All these characteristics are consistent with the conclusions of Fig. 1.

## The FFLO Superfluid with a Beat in the 2D WSM

For the WSM, Fermi surface is only four Weyl points at different momenta. This anomalous band structure provides the natural basis for the emergence of novel and inhomogeneous superfluid states, especially for the FFLO state^46,47^. Here, with contact attractive interaction, Fermi gas forms s-wave superfluids. We define the order parameter as Δ_*i*_ = −*U*_*int*_〈*c*_*i*↓_*c*_*i*↑_〉 with the interaction strength *U*_*int*_ = 5. ${H}_{\Delta }=\sum _{i}\,({\Delta }_{i}{c}_{i\uparrow }^{\dagger }{c}_{i\downarrow }^{\dagger }+{\Delta }_{i}^{\ast }{c}_{i\downarrow }{c}_{i\uparrow })$. The all Hamiltonian is *H*_*sc*_ = *H* + *H*_Δ_ combining with *H*_Δ_ and Eq. (1), which can be diagonalized by performing canonical tranformations: ${c}_{i\sigma }=\sum _{n}\,{u}_{i\sigma }^{n}{\gamma }_{n}+{v}_{i\sigma }^{n\ast }{\gamma }_{n}^{\dagger }$. We can obtain self-consistency BdG Eq as: $[{\gamma }_{n}^{(\dagger )},{H}_{sc}]=(\,-\,){E}_{n}{\gamma }_{n}^{(\dagger )}$. The real-space order parameters Δ_*i*_ are determined self-consistently for a fixed chemical potential.

**Figure 4:**
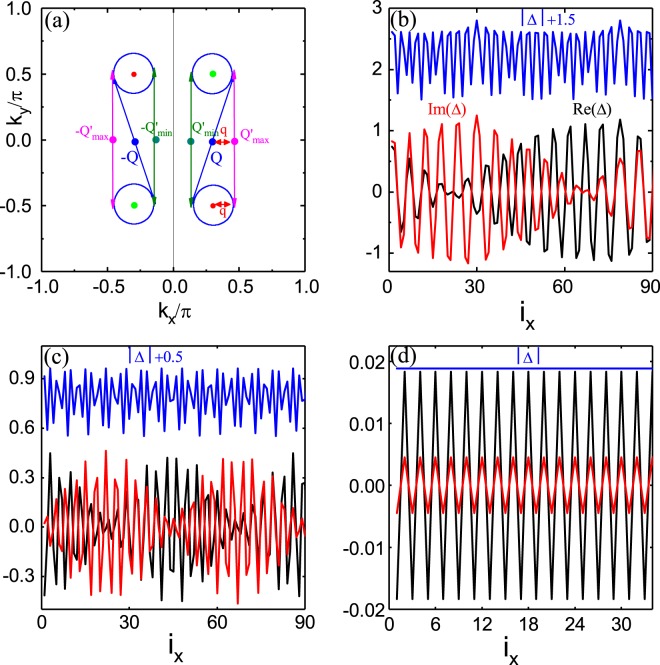
The schematic diagram and the FFLO order parameters for different *h*_*x*_. (**a**) The schematic diagram of the Fermi surface. The order parameters of the LO state with a beat at *h*_*x*_ = 1.1 for (**b**) and with two beat at *h*_*x*_ = 1.55 for (**c**). (**d**) The order parameter of the FF state at *h*_*x*_ = 2 with *π* phase. Other parameters are *t* = 1 as energy unit.

To distinguish the source of different modulations for inhomogeneous superfluid states, such as the FFLO state with a beat, we show the schematic diagram of the Fermi surface under the superfluid states in Fig. 4(a). Because of the unusual Fermi surface in the WSM, the Fermi surface of the superfluid state is represented by four blue circles, one of which is around the position of the Weyl point in the first RBZ. Our calculations show that the Δ_*i*_ is uniform along *y* direction, and the finite center-of-mass momentum ±*Q* of the cooper pairs only exist along *x* direction, marking the so-called Larkin-Ovchinnikov(LO) state(a spatially varying order parameter amplitude). It implies that the cooper pairs only occur between up and down Fermi surfaces being parallel to *k*_*y*_ direction in Fig. 4(a), and cannot occur between left and right Fermi surfaces along *k*_*x*_ direction. In particular, for the two Fermi surfaces parallel to *k*_*y*_ direction, there exist two kinds of pairs. If the atoms from the upper and lower Fermi surfaces form the cooper pair along the diagonal direction, it holds the finite center-of-mass momentum $Q$, which is so-called Larkin-Ovchinnikov(LO) state^47^. It is labeled by the blue double arrow, such as shown in Fig. 4(a). If they form the cooper pair perpendicular to the *k*_*x*_ axis, their finite center-of-mass momenta can distribute in the interval $[\,\pm \,{Q^{\prime} }_{min},\pm \,{Q^{\prime} }_{max}]$, and ${Q^{\prime} }_{min}$ and ${Q^{\prime} }_{max}$ are labeled by the green and magenta double arrows respectively. The maximum of the difference of the *Q*′ is (${Q^{\prime} }_{max}-{Q^{\prime} }_{min}$) = 2*q*, *q* is roughly equal to the radius of the Fermi surface. Once the cooper pairs with the different ±*Q* and ±*Q*′ emerge, the LO states with different frequencies (*Q* and *Q*′) overlap each other to produce beating pattern such as in Fig. 4(b,c). From Fig. 4(b), we can only observe a beat in the system of specific size when *h*_*x*_ = 1.1. When *h*_*x*_ = 1.55 in Fig. 4(c), the Δ_*i*_ can be reduced, Fermi surface of the LO state shrink accordingly, which results in the reduction of the difference *q*′ of *Q*′ and *Q* simultaneously. This relatively increases the value of *q*′/*Q*, so one beat has evolved into two beats. When *h*_*x*_ = 2 such as in Fig. 4(d), the difference *q*′ of *Q*′ also becomes negligible. From Fig. 1(g), we can find that the finite center-of-mass momentum *Q* is only *π*, so this inhomogeneous superfluid state is the *π*-Fulde-Ferrell(FF) state (a phase-modulated order parameter with a uniform amplitude)^46^. All the above novel superfluid states can be determined by the special structure of the Fermi surface.

## Summary

In summary, we theoretically propose and study the 2D WSM with cold atoms in a square optical lattice subjected to the non-Abelian gauge field, and successfully identify the Weyl points and their evolutions by the single-particle spectra and the Bloch Zener Oscillation technique under different *h*_*x*_. The Fermi velocities and Berry connections of the 2D WSM are manifestly uncovered with the change of the magnetic field. Moreover, based on the particular structure of the Fermi surface in the 2D WSM, we examine the superfluidity of attractive Fermi gases, and the FFLO states with different beats are revealed, which significantly enriches and deepens our awareness of the novel and inhomogeneous superfluid states. The 2D WSM combining with the 3D WSM provides a promising platform for exploring the exotic physics of WSMs. Considering that all the ingredients to observe the 2D Weyl points in the optical lattice have been achieved in recent experiments, we expect that the 2D WSM will be realized in the future experiments.

## References

[1] Hasan MZ, Kane CL (2010). Colloquium: Topological insulators. Rev. Mod. Phys..

[2] Qi X, Zhang S (2011). Topological insulators and superconductors. Rev. Mod. Phys..

[3] Wan X, Turner AM, Vishwanath A, Savrasov SY (2011). Topological semimetal and Fermi-arc surface states in the electronic structure of pyrochlore iridates. Phys. Rev. B.

[4] Xu G, Weng H, Wang Z, Dai X, Fang Z (2011). Chern Semimetal and the Quantized Anomalous Hall Effect in HgCr_2_Se_4_. Phys. Rev. Lett..

[5] Burkov AA, Balents L (2011). Weyl Semimetal in a Topological Insulator Multilayer. Phys. Rev. Lett..

[6] Young SM (2012). Dirac Semimetal in Three Dimensions. Phys. Rev. Lett..

[7] Liu ZK (2014). Discovery of a Three-Dimensional Topological Dirac Semimetal, Na_3_Bi. Science.

[8] Huang S-M (2015). A Weyl Fermion semimetal with surface Fermi arcs in the transition metal monopnictide TaAs class. Nature Commun.

[9] Weng H, Fang C, Fang Z, Bernevig A, Dai X (2015). Weyl Semimetal Phase in Noncentrosymmetric Transition-Metal Monophosphides. Phys. Rev. X.

[10] Lu L (2015). Experimental observation of Weyl points. Science.

[11] Xu S-Y (2015). Discovery of a Weyl fermion semimetal and topological Fermi arcs. Science.

[12] Lv BQ (2015). Experimental Discovery of Weyl Semimetal TaAs. Phys. Rev. X.

[13] Soluyanov AA (2015). Type-II Weyl semimetals. Nature.

[14] Liang, A. *et al*. Electronic Evidence for Type II Weyl Semimetal State in MoTe_2_. *arXiv*:1604.01706.

[15] Jiang J (2017). Signature of type-II Weyl semimetal phase in MoTe_2_. Nat. Commun..

[16] Deng K (2016). Experimental observation of topological Fermi arcs in type-II Weyl semimetal MoTe_2_. Nature Physics.

[17] Xu, S.-Y. *et al*. Discovery of Lorentz-violating Weyl fermion semimetal state in LaAlGe materials. *arXiv*:1603.07318.

[18] Huang L (2016). Spectroscopic evidence for a type II Weyl semimetallic state in MoTe_2_. Nature Materials.

[19] Zyuzin AA, Burkov AA (2012). Topological response in Weyl semimetals and the chiral anomaly. Phys. Rev. B.

[20] Wang Z, Zhang S-C (2013). Chiral anomaly, charge density waves, and axion strings from Weyl semimetals. Phys. Rev. B.

[21] Parameswaran SA, Grover T, Abanin DA, Pesin DA, Vishwanath A (2014). Probing the Chiral Anomaly with Nonlocal Transport in Three-Dimensional Topological Semimetals. Phys. Rev. X.

[22] Yu Z, Yao Y, Yang SA (2016). Predicted Unusual Magnetoresponse in Type-II Weyl Semimetals. Phys. Rev. Lett..

[23] Udagawa M, Bergholtz EJ (2016). Field-Selective Anomaly and Chiral Mode Reversal in Type-II Weyl Materials. Phys. Rev. Lett..

[24] Lu L, Fu L, Joannopoulos JD, Soljačić M (2013). Weyl points and line nodes in gyroid photonic crystals. Nature photonics.

[25] Isobe H, Nagaosa N (2016). Coulomb Interaction Effect in Weyl Fermions with Tilted Energy Dispersion in Two Dimensions. Phys. Rev. Lett..

[26] Hao L, Ting CS (2016). Topological phase transitions and a two-dimensionalWeyl superconductor in a half-metal/superconductor heterostructure. Phys. Rev. B.

[27] Huang L (2016). Experimental realization of two-dimensional synthetic spinCorbit coupling in ultracold Fermi gases. Nature. Phys.

[28] Wu Z (2016). Realization of two-dimensional spin-orbit coupling for Bose-Einstein condensates. Science.

[29] Jiang JH (2012). Tunable topological Weyl semimetal from simple-cubic lattices with staggered fluxes. Phys. Rev. A.

[30] Dubček T (2015). Weyl Points in Three-Dimensional Optical Lattices: Synthetic Magnetic Monopoles in Momentum Space. Phys. Rev. Lett..

[31] He W-Y, Zhang S, Law KT (2016). Realization and detection of Weyl semimetals and the chiral anomaly in cold atomic systems. Phys. Rev. A.

[32] Xu, Y. & Lu-Ming, D. Type-II Weyl Points in Three-Dimensional Cold Atom Optical Lattices. *arXiv*:1607.04924.

[33] Xu Y, Zhang F, Zhang C (2015). Structured Weyl Points in Spin-Orbit Coupled Fermionic Superfluids. Phys. Rev. Lett..

[34] Ganeshan S, Das Sarma S (2015). Constructing a Weyl semimetal by stacking one-dimensional topological phases. Phys. Rev. B.

[35] Lin. YJ (2009). Bose-Einstein Condensate in a Uniform Light-Induced Vector Potential. Phys. Rev. Lett..

[36] Lin YJ, Compton RL, Jiménez-Garca K, Porto JV, Spielman IB (2009). Synthetic magnetic fields for ultracold neutral atoms. Nature.

[37] Lin YJ, Jiménez-Garca K, Spielman IB (2011). SpinCorbit-coupled BoseCEinstein condensates. Nature.

[38] Xiao D, Chang M-C, Niu Q (2010). Berry phase effects on electronic properties. Rev. Mod. Phys..

[39] Dalibard J, Gerbier F, Juzeliūnas G, Öhberg P (2011). Colloquium: Artificial gauge potentials for neutral atoms. Rev. Mod. Phys..

[40] Yang SA, Pan H, Zhang F (2009). Dirac and Weyl Superconductors in Three Dimensions. Phys. Rev. Lett..

[41] Zhao YX, Schnyder AP, Wang ZD (2016). Unified Theory of *PT* and *CP* Invariant Topological Metals and Nodal Superconductors. Phys. Rev. Lett..

[42] Sun FD, Yu XL, Ye JW, Fan H, Liu WM (2013). Topological Quantum Phase Transition in Synthetic Non-Abelian Gauge Potential:Gauge Invariance and Experimental Detections. Sci. Rep.

[43] Lim LK, Fuchs JN, Montambaux G (2012). Bloch-Zener Oscillations across a Merging Transition of Dirac Points. Phys. Rev. Lett..

[44] Tarruell L (2012). Creating, moving and merging Dirac points with a Fermi gas in a tunable honeycomb lattice. Nature.

[45] Zhang DW, Zhu SL, Wang ZD (2015). Simulating and exploringWeyl semimetal physics with cold atoms in a two-dimensional optical lattice. Phys. Rev. A.

[46] Fulde P, Ferrell RA (1964). Superconductivity in a strong spin exchange field. Phys. Rev.

[47] Larkin AI, Ovchinnikov YN (1964). Nonuniform state of superconductors. Zh. Eksp. Teor. Fiz.

